# Missing upper incisors: a retrospective study of orthodontic space closure versus implant

**DOI:** 10.1186/s40510-015-0072-2

**Published:** 2015-02-25

**Authors:** Abdolreza Jamilian, Letizia Perillo, Marco Rosa

**Affiliations:** Department of Orthodontics, Dental Branch, Craniomaxillofacial Center Islamic Azad University, No 2713, Vali Asr St., Tehran, 1966843133 Iran; Post Graduate Orthodontic Program, Department of Orthodontics, Second University of Naples, Via bellini, 44 80135, Naples, Italy; Post-graduate School in Orthodontics, Insubria University, P.za della Mostra, 19 38122, Trento, Italy

**Keywords:** Implant, Orthodontic space closure, Missing maxillary incisor, Infraocclusion, Periodontal health

## Abstract

**Background:**

The aim of this retrospective study was to compare the esthetic, periodontal, and functional outcomes of orthodontic space closure versus implant substitution in patients with missing maxillary incisors 5 years after completion of treatment.

**Methods:**

The study group consisted of ten patients treated with orthodontic space closure (six males, four females, mean age 19 ± 2.1 years at the completion of treatment) and ten patients treated with implant insertion (five males, five females, mean age 20 ± 1.4 years at the time of implant insertion). Tooth mobility, plaque index, probing depth, infraocclusion, open gingival embrasure (black triangle), and temporomandibular joint function were recorded at the 5.6 years follow-up. Self-perceived dental esthetic appearance was also evaluated through a visual analog scale (VAS) questionnaire. *T*-test was used to evaluate the data.

**Results:**

All patients were equally satisfied with the appearance of their teeth 5.6 ± 0.4 years after the completion of treatment. No statistically significant differences were found in relation to the VAS scores of the subjects (*P* < 0.857). No significant differences were found in tooth mobility, plaque index (*P* < 0.632), and the prevalence of signs and symptoms of temporomandibular disorders. However, significant infraocclusion was noticed in all implant patients (*P* < 0.001). Probing depth was also significantly higher in implant patients (*P* < 0.001).

**Conclusions:**

Orthodontic space closure and implant of missing maxillary incisors produced similar, well-accepted esthetic results. None of the treatments impaired temporomandibular joint function. Nevertheless, infraocclusion was evident in implant patients. Space closure patients also showed better periodontal health in comparison with implant patients.

## Background

The absence of upper incisors is a serious problem and often needs a challenging treatment. The treatment alternatives of missing upper incisors include orthodontic space closure, resin-bonded bridgework, osseointegrated implants, removable partial dentures, and autotransplantation of developing premolars [1-7]. Although each of these methods is a viable treatment option, implant insertion and space closure are more popular among clinicians.

Implant substitution is considered an optimal solution considering the possibility of obtaining an ideal occlusion and the indisputable advantage of avoiding any damage to the adjacent teeth [1,8]. Orthodontic space closure, by mesial movement of the adjacent teeth, also provides satisfactory esthetic and functional long-term results [9,5,10]. One of the major advantages of space closure is that treatment is finished immediately after orthodontics and, in the case of adolescents, there is no necessity of waiting years until the ‘end of growth’ to replace the missing tooth. Moreover, the result is natural and all the changes in the long term will also be natural, unlike what could happen in the presence of a foreign body.

The aim of this study was to examine and compare, 5 years post treatment, the esthetic outcomes, function, and periodontal health in two groups of subjects with one or two missing maxillary incisors and treated either with implant substitution or orthodontic space closure.

## Methods

This retrospective observational study was carried out in accordance with the ethical standards set forth in the 1964 Declaration of Helsinki. Informed written consent was obtained from each patient and a parent or guardian.

Twenty-six patients with missing maxillary incisors, who were treated by one orthodontist between 2004 and 2006 in two centers, were all invited to a follow-up examination. One of the patients was omitted from the study because she could have only been treated by means of implant insertion. Five of the patients refused to attend. Out of the 20 remaining patients, 10 were treated by implant insertion and 10 by orthodontic space closure.

The selection criteria included patients who had the following:

- Congenital absence of the maxillary central and/or lateral incisor.
- Patients treated with orthodontic space closure and/or implant.
- Patients who could have been treated by either space closure or implant insertion.
- Patients not treated with autotransplantation or crown bridge.
- No periodontal breakdown or history of periodontal disease.

The implant group (IG) consisted of ten patients (five males, five females, mean age 20 ± 1.4 years at the time of implant insertion). All the implants were of the same type and were inserted by the same surgeon. Fourteen teeth including four lateral and ten central maxillary incisors were totally missing in the implant group. The average treatment time of these patients was 18 ± 4 months. The orthodontic space closure (OSC) group consisted of ten patients (six males, four females, mean age 19 ± 2.1 years at the completion of treatment). Seventeen teeth including seven lateral and ten central maxillary incisors were totally missing in the OSC group. The average treatment time of these patients was 29 ± 7 months (Table 1). For retention, upper and lower Hawley retainers were worn by all the patients for 1 year.

**Table 1:** **Distribution of age, sex, and left or right missing teeth in the OSC and implant groups**

**Treatment group**		**OSC group**	**Implant group**	**Significance**
Age at the time of treatment completion		19 ± 2.1	20 ± 1.4	NS
Gender	Male	6	5	NS
	Female	4	5	NS
Lateral incisor	Left	3	1	NS
	Right	4	3	NS
Central incisor	Left	4	4	NS
	Right	6	6	NS
Treatment time (months)		29 ± 7	18 ± 4	*P* < 0.001

OSC, orthodontic space closure; NS, not significant.

The follow-up records were taken 5 years or more after the completion of treatment (mean interval was 5.6 ± 0.4 years).

Tooth mobility (TM) [11], plaque index (PI) [12,13], probing depth (PD), infraocclusion (I), open gingival embrasure (black triangle) [14], and temporomandibular joint dysfunction (TMD) of the patients were recorded at the follow-up examinations (Table 2).

**Table 2:** **Clinical records of periodontal states**

**Index**	**Assessment method**
Tooth mobility	Mobility more than 1 mm was recorded
Probing depth	Pockets exceeding 3 mm were recorded
Infraocclusion	Infraocclusion exceeding 1 mm was recorded
TMD	Tooth clenching, grinding, and TMJ sounds were recorded

TMD, temporomandibular joint dysfunction; TMJ, temporomandibular joint.

**Figure 1:**
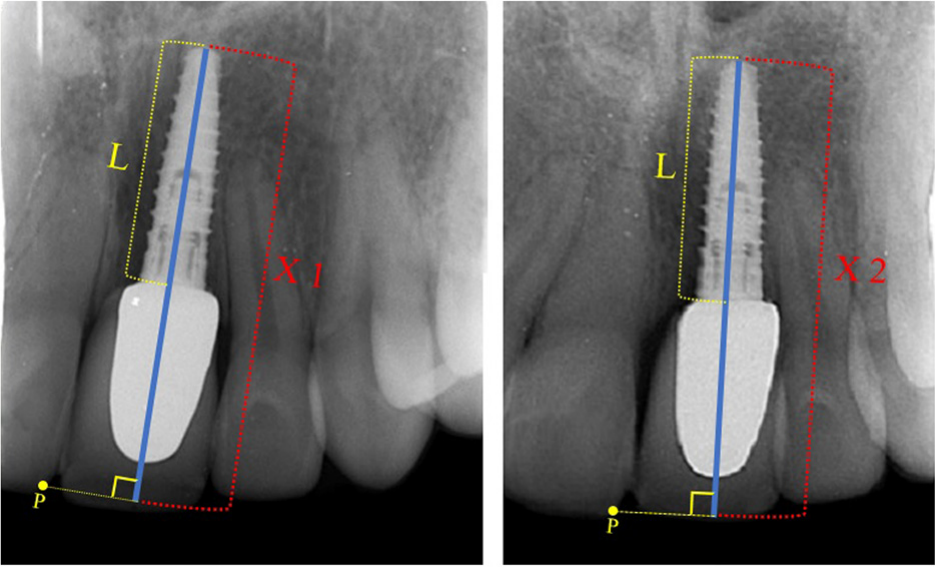
**Method of measuring infraocclusion.**

Probing depth was recorded at four sites for each upper central and lateral incisor with a periodontal probe. Identical intra-oral radiographs, taken with the ‘parallel technique’, were used for assessment of infraocclusion. Evaluation of the image distortion was done by measuring the length of the implant (L) as it appears on the radiography and comparing it to the real length of the implant (Figure 1). In order to evaluate infraocclusion, a reference point (p) was selected on the intersection between the incisal and mesial borders of the tooth adjacent to the implant. Afterwards, a line was drawn from this point perpendicular to the longitudinal axis of the implant creating the projection of p. The distance between the projection of p and the apex of the implant was measured, and the difference between this distance after the completion of treatment and follow-up examination was recorded as infraocclusion (*X*2 − *X*1) (Figure 1). The distance of the alveolar crest to the contact point of the teeth was used to evaluate the presence of an open embrasure (black triangle). A distance of more than 5 mm was considered as black triangle [14].

The prevalence of signs and symptoms of TMD was assessed by means of an anamnestic questionnaire [15] (Table 3). A score ‘0’ indicated the absence of symptoms, whereas ‘1’ was given for a report of an occasional occurrence. Grade 2 indicated the presence of symptoms, and a score of ‘3’ was used to indicate severe pain or bilateral symptoms. The sum of the scores was used to classify the sample into four categories: (1) from 0 to 3, TMD free; (2) from 4 to 8, mild TMD; (3) from 9 to 14, moderate TMD; and (4) from 15 to 23, severe TMD. Plaque was measured by the plaque index developed by Quigley and Hein [12] and modified by Turesky et al. [13]. The scoring system is as follows: 0 = no plaque, 1 = separate flecks of plaque at the cervical margin of the tooth, 2 = a thin continuous band of plaque at the cervical margin, 3 = a band of plaque wider than 1 mm but covering less than one third of the crown, 4 = plaque covering at least one third but less than two thirds of the crown, 5 = plaque covering two thirds or more of the crown. None of the patients had systemic diseases or long-term pharmacological treatment until the end of follow-up studies.

**Table 3:** **Questionnaire for assessing TMD**

**Severity of occurrence**	**0**	**1**	**2**	**3**
1. Do you have difficulty in opening your mouth?				
2. Do you have difficulty in moving or using your jaw?				
3. Do you have tenderness or muscular pain when chewing?				
4. Do you have frequent headaches?				
5. Do you have neck aches or shoulder pain?				
6. Do you have pain in or about the ears?				
7. Are you aware of noises in the jaw joints?				
8. Do you consider your bite “normal”?				
9. Do you use only one side of your mouth when chewing?				
10. Do you have morning facial pain?				

Patient satisfaction was assessed using the visual analog scale (VAS) [16,17]. The subjects were asked to record their happiness with their dental and facial appearance on a 10-cm VAS having phrases ‘very dissatisfied’ (score 0) on the left end and ‘very satisfied’ (score 10) on the right end.

Each patient was examined by two examiners in two sessions with an interval of 1 week. The inter-examiner agreement was calculated through kappa analysis. A kappa value of 0.96 showed perfect agreement. *T*-test was used to evaluate the data of the study.

## Results

Tables 4 and 5 show the results 5.6 ± 0.4 years after treatment. Increased mobility was noticed in only one implant, but no tooth mobility was present in any of the OSC patients. More than 3 mm increase of probing depth was found in 12 implants, while it increased more than 3 mm in only 3 teeth in the OSC group (*P* < 0.001). The vertical step measured on radiographs of implant patients varied between 1.2 and 1.6 mm, which showed that all implant-supported teeth had increased infraocclusion of more than 1 mm, while none of the OSC patients showed infraocclusion (*P* < 0.001) (Figure 1). Black triangle was seen in one of the implant patients and three of the OSC patients.

**Table 4:** **Comparison of parameters between implant and space closure groups**

**Recorded scores**	**Tooth mobility (number of teeth)**	**Probing depth (number of teeth)**	**Infraocclusion (number of teeth)**	**Plaque index (mean ± SD)**	**TMD (mean ± SD)**
Implant group	1	12	14	3.1 ± 1.2	1.3 ± 1.1
OSC group	0	3	0	2.9 ± 1.1	1.5 ± 1.03
*P* value	NS	0.001*	0.001*	0.632	0.605

*Level of significance set at *P* < 0.05. TMD, temporomandibular joint dysfunction; SD, standard deviation; OSC, orthodontic space closure.

**Table 5:** **Patient satisfaction assessed by visual analog scale (VAS) after 5 years since the completion of treatment**

	**Implant group**	**OSC group**	***P*** **value**
	**Mean ± SD**	**Mean ± SD**	
VAS scores	8.7 ± 1.3	8.8 ± 1.2	0.857

OSC, orthodontic space closure; SD, standard deviation.

No signs and symptoms of temporomandibular joint dysfunction including history of headache, locking, difficulty in opening/closing mouth, and tooth clenching were noticed in any of the patients, and *T*-test showed that there were no statistically significant differences between the two groups (*P* < 0.605). Five years after the completion of treatment, the plaque indexes of the implant and OSC patients were 3.1 ± 1.2 and 2.9 ± 1.1, respectively (*P* < 0.632) (Table 4).

VAS scores of 8.7 ± 1.3 and 8.8 ± 1.2 were recorded for the implant and OSC patients, subjectively. The scores which ranged from 7 to 10 showed that both groups were almost equally satisfied with the appearance of their teeth after 5 years. *T*-test did not show any statistically significant difference between the two groups (Table 5).

**Figure 2:**
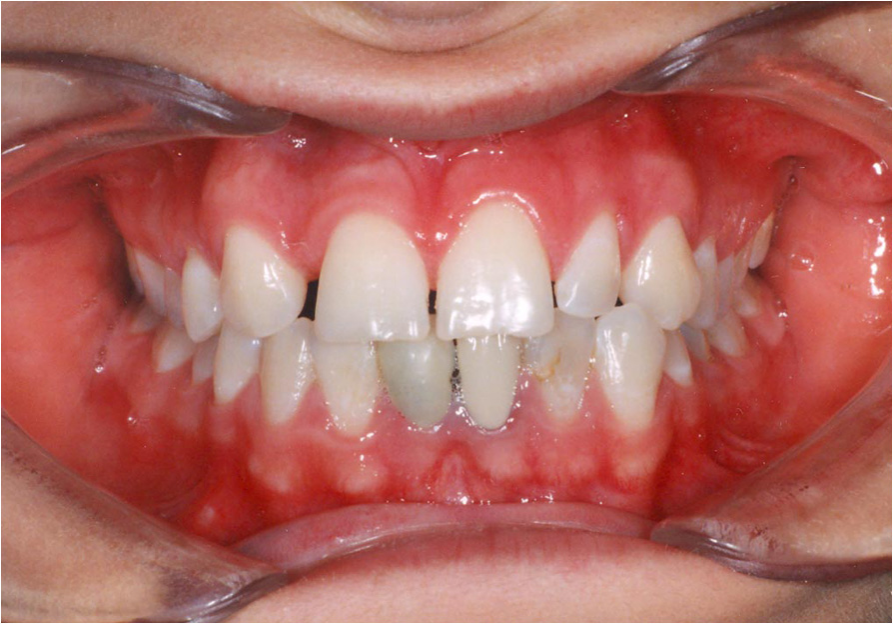
**A patient with a missing maxillary lateral incisor.**

**Figure 3:**
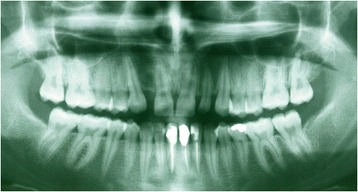
**Panoramic radiograph of the same patient with a missing maxillary lateral incisor.**

**Figure 4:**
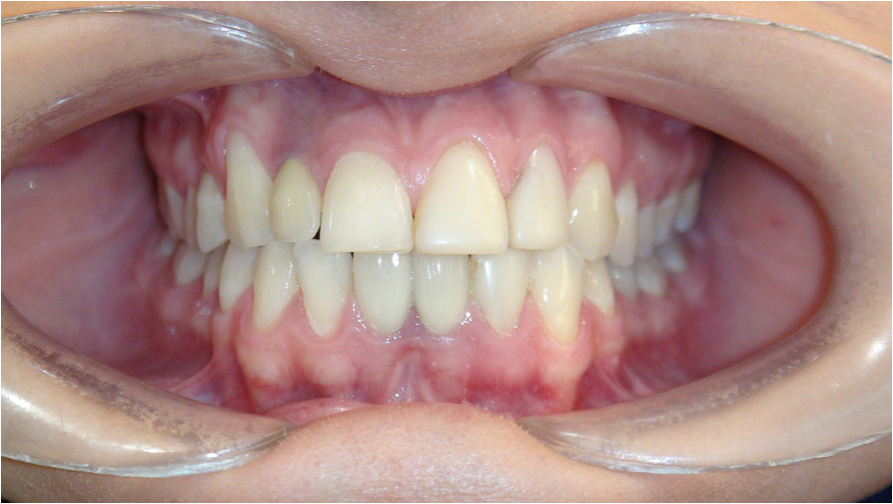
**The same patient 5 years after implant insertion.**

**Figure 5:**
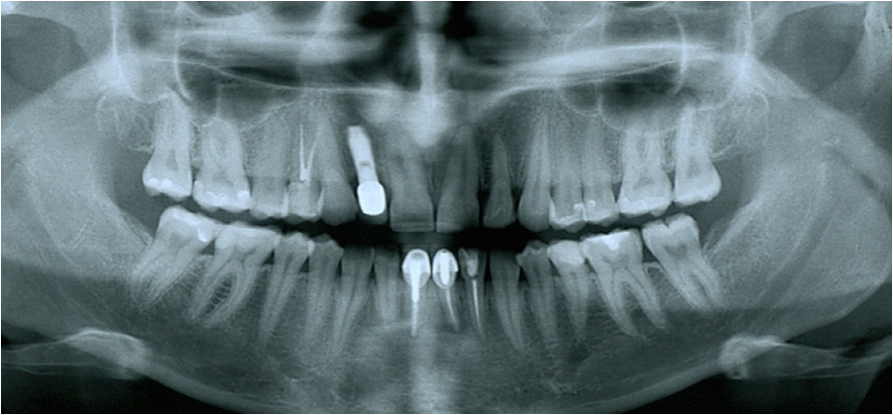
**Panoramic radiograph of the same patient 5 years after implant insertion.**

**Figure 6:**
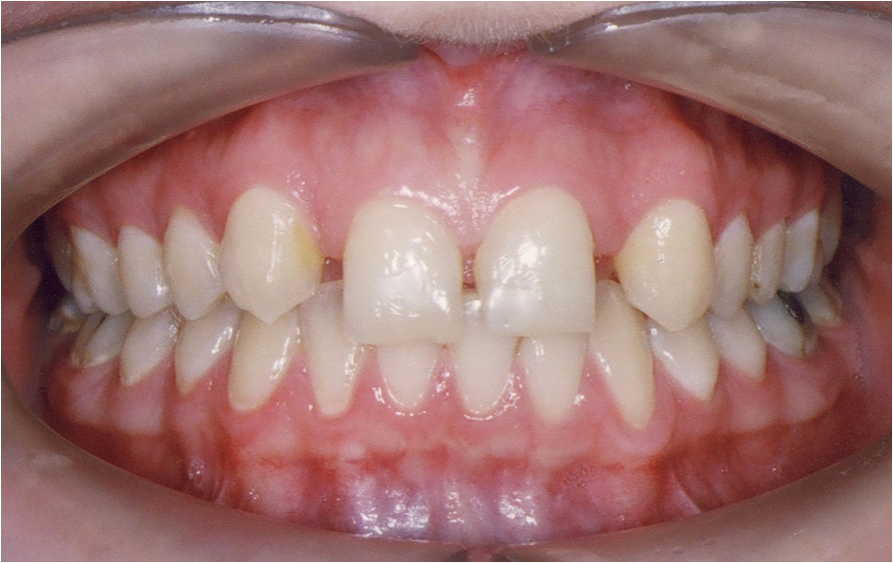
**A patient with missing maxillary lateral incisors.**

**Figure 7:**
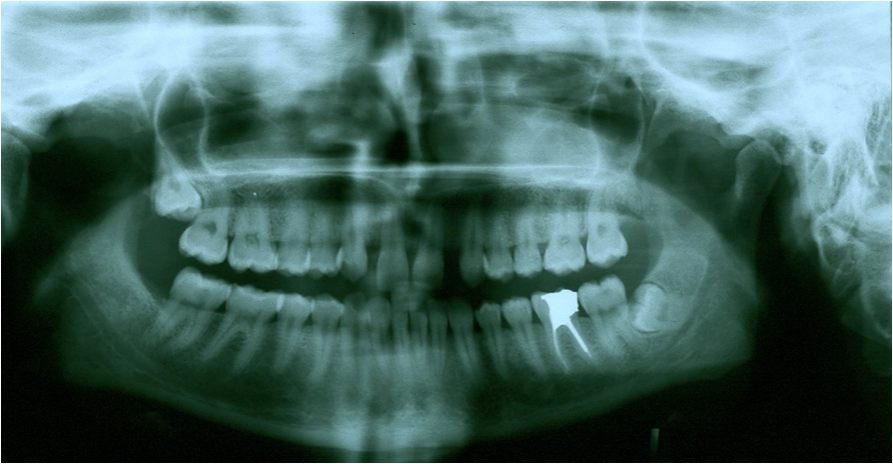
**Panoramic radiograph of the same patient with missing maxillary lateral incisors.**

**Figure 8:**
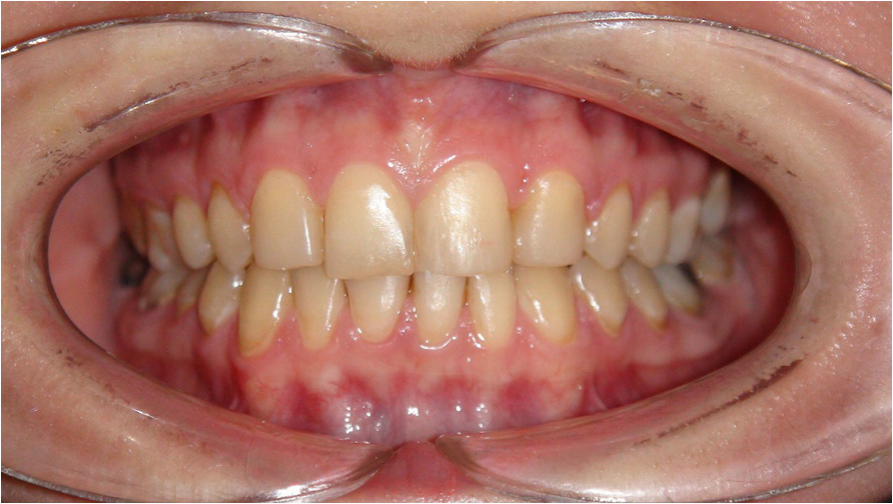
**The same patient 5 years after orthodontic space closure.**

**Figure 9:**
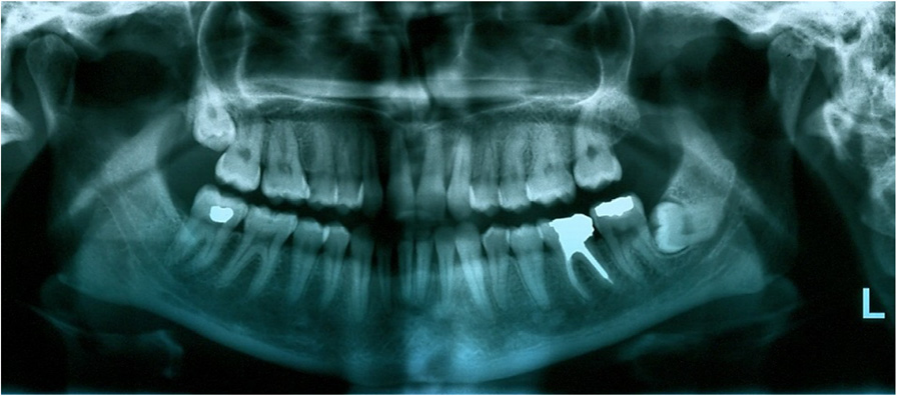
**Panoramic radiograph of the same patient 5 years after orthodontic space closure.**

Figures 2 and 3 show a patient with a missing upper lateral incisor, and Figures 4 and 5 show the same patient 5 years after implant insertion. Figures 6 and 7 show a patient with missing maxillary lateral incisors treated by orthodontic space closure, and Figures 8 and 9 show the same patient 5 years after the completion of treatment.

## Discussion

The present retrospective study shows that both treatment modalities resulted in satisfactory and well-accepted esthetic outcomes after 5 years. However, infraocclusion and increased probing depth were seen in the implant patients.

These findings correspond to the findings of Bernard et al. [18], Kuijpers et al. [19], and Jemt et al. [20] who reported that even if the implant is inserted after 19 years of age, the adjacent teeth and surrounding alveolar bone may continue to develop vertically and may continue to erupt resulting in infraocclusion of the implant restoration, i.e., a discrepancy between the gingival margin of the implant restoration and the gingival margin of the adjacent natural teeth may appear in a few years after treatment, and the implant becomes submerged. In cases where the upper and lower incisors are not in contact, the resulted extrusion might be 0/2 to 0/3 mm per year. Implants behave like ankylosed teeth, and their position cannot change in contrast to their neighboring natural teeth; therefore, even small tooth movements of adjacent teeth after implant placement can cause esthetic problems [21-23]. The disharmonious levels of the gingival margins resulting from infra-positioned implant restorations are an evident disadvantage in patients with a high smile line. Thus, ‘gummy smile’, or patients who show the gingival margins, is in our opinion a contraindication for implant substitution of the upper incisors [5].

Implant substitution could have other disadvantages. It has been shown that most implant crowns show some lack of interdental papillary fill, particularly on the distal papilla [24]. Furthermore, blue coloring of the labial gingiva has been reported in above more than 50% of single-implant crowns at 4-year follow-ups [25]. Peri-implantitis, gingivitis, increased probing depth, bleeding on probing, and progressive loss of marginal bone support at the buccal aspect of the implant have also been reported in implants [22,24,26,27].

Finally, the biggest disadvantage of the implant alternative is that adolescents must wait many years after orthodontic treatment before the implant insertion. During this interim time, patients must use temporary restorations that often create many different problems and replacements. Nevertheless, the comparatively shorter and easier treatment makes implants a favorable treatment option for substituting missing teeth.

In contrast to the abovementioned disadvantages related to implants, orthodontic space closure is a viable and safe procedure that could provide better long-term results [9]. Moreover, none of the abovementioned disadvantages have been noticed in orthodontic space closure [28,29]. Nevertheless, the applicability of orthodontic space closure for missing incisors is sometimes questioned. Concerns may be expressed related to the treatment complexity, the risk for space reopening, the questioned increased functional load on the first premolar roots, and the quality of the esthetic result especially in the case of a lateral incisor’s root supporting the larger crown of a central incisor [30].

The main advantages of the space closure alternative compared to implants can be highlighted as follows:

- Treatment is finished immediately after orthodontics. This is for crucial interest when treating adolescent patients.
- Periodontal problems will not develop in space closure because the tooth has moved along with its bone and surrounding tissues.
- Space closure provides patients with better long-term esthetic results in the transition area, due to lack of bone loss and periodontal problems.
- Prosthetic replacement of the lost incisor also by partial denture or bonded bridges could need further treatments to replace the restorations or to fix possible periodontal breakdown.

Allocation of patients in this study and comparing their treatment effects could be considered as one of the weaknesses of the current study because the patients were allocated to the implant or the OSC group based on the space between their teeth. However, it should be considered that the results of the current study can assist in treatment planning for borderline cases who could be treated by either implant insertion or orthodontic space closure.

## Conclusions

Five years after treatment, orthodontic space closure and implant substitution of missing maxillary incisors produced similar satisfactory esthetic results. Neither of the treatments impaired temporomandibular joint function. However, orthodontic space closure patients had better periodontal health in comparison with implant substitution patients. Furthermore, infraocclusion more than 1 mm was noticed in all the implant patients.
